# Supplementary material for: A set of multi-entry identification keys to African frugivorous flies (Diptera, Tephritidae)
Source: Zookeys. 2014 Jul 24;(428):97–108. doi: 10.3897/zookeys.428.7366 (PMC4143993; doi:10.3897/zookeys.428.7366)
Supplement: Supplementary material 6 — Key to Ceratitis [file zookeys-428-097-s006.zip › SF6_ZooKeys_key to Ceratitis/key/SF6_ZooKeys_key to Ceratitis/Media/Html/Ceratitis ovalis .htm]

Ceratitis (Ceratalaspis) ovalis Munro


***Ceratitis*** **(*Ceratalaspis*)
*ovalis* Munro**

*Ceratitis* (*Pardalaspis*) *ovalis*
Munro, 1935: 310.

Body
length: 4.00 mm; wing length: 4.40 mm.

Male

Head.
Antenna yellow-orange. Third antennal segment twice to three times as long as
second segment. Arista distinctly plumose, with very long hairs over entire
length. Frons with short scattered hairs which are largely the same colour as
frons; convex, in lateral view not distinctly projecting forwards at antennal
implant. Chaetotaxy normal for subgenus.

Thorax.
Postpronotum white, with black spot. Ground colour of mesonotum pale with
orange tinge. Chaetotaxy normal for subgenus. Scapular setae obscured in
holotype. One anepisternal bristle. Scutellum yellow with three black separate
markings apically; basally with two dark spots joining. Subscutellum completely
dark.

Legs
yellow; setation typical for subgenus, mainly pale especially on femora.
Posterior and posterodorsal rows on fore femur pale.

Wing
bands with extensive yellow markings. Banding, marginal band continuous;
cubital band free; medial band absent; crossvein r-m at middle of discal cell.
Crossvein dm-cu posteriorly more inwards than anteriorly. cup extension only
weakly sinoid.

Abdomen.
Orange-brown colour. Setation and banding typical for subgenus.

Female

Unknown.

(Description
after De Meyer, 1998)
